# Multifarious activities of cellulose degrading bacteria from Koala (*Phascolarctos cinereus*) faeces

**DOI:** 10.1186/s40781-015-0056-2

**Published:** 2015-07-08

**Authors:** Surender Singh, Palanisami Thavamani, Mallavarapu Megharaj, Ravi Naidu

**Affiliations:** Division of Microbiology, Indian Agricultural Research Institute, New Delhi, 110012 India; University of Newcastle, Callaghan, New South Wales Australia; formerly, Centre for Environmental Risk Assessment and Remediation (CERAR), University of South Australia, Mawson Lakes, Adelaide, South Australia Australia; Cooperative Research Centre for Contamination Assessment and Remediation of the Environment (CRC CARE), Adelaide, 5095 South Australia Australia

**Keywords:** Koala, cellulase, enzymes, biofuel, phenanthrene

## Abstract

Cellulose degrading bacteria from koala faeces were isolated using caboxymethylcellulose-Congo red agar, screened *in vitro* for different hydrolytic enzyme activities and phylogenetically characterized using molecular tools. *Bacillus* sp. and *Pseudomonas* sp. were the most prominent bacteria from koala faeces. The isolates demonstrated good xylanase, amylase, lipase, protease, tannase and lignin peroxidase activities apart from endoglucanase activity. Furthermore many isolates grew in the presence of phenanthrene, indicating their probable application for bioremediation. Potential isolates can be exploited further for industrial enzyme production or in bioremediation of contaminated sites.

## Background

The koala (*Phascolarctos cinereus*) is an arboreal herbivorous marsupial native to Australia and they feed almost exclusively on eucalyptus leaves. Eucalyptus leaves are low in protein, high in recalcitrant substances like lignin, cellulose, tannin and essential oils that are toxic to most species [1, 2]. Both aerobic and anaerobic bacteria are the main degraders of eucalyptus leaves in the hindgut of koala [3] and this process requires the activity of cellulases, hemicellulases, ligninases, tannase and other enzymes that are of high biotechnological interest. Intensive studies have been undertaken on the gut microflora of the koala [4–6]. Osawa et al. [7] and Nemoto et al. [8] reported that groups of streptococci and enterobacteria, both of which could degrade the protein complexed with tannin, were the most predominant members of koalas’ facultative anaerobic faecal flora. Recently, Peterson et al. [9, 10] isolated a number of fungi from koala faeces indicating diverse enzyme activities against recalcitrant substrates such as cellulose and lignin. However, little work has been done on trying to understand the genetic and functional diversity of faecal bacteria other than tannin degraders like cellulose degrading ones. In recent times bacterial cellulases have garnered attention due to their robust ability to inhabit a wide variety of extreme environments, and the enzymes produced by these are stable in such harsh environments during various industrial applications [11]. This means that the processing costs can be cut.

PCR-based DNA-typing methods like 16S rRNA gene sequencing for culturable bacteria are currently universally applicable, simple and rapid. In the meantime denaturing gradient gel electrophoresis (DGGE) coupled with sequence analysis of 16S rRNA gene has emerged as a useful technique for studying unculturable microorganisms from environmental samples [12]. However, these molecular techniques do not explain the functional activities of the microorganisms in different biological niches. Consequently in this study we have made an attempt to understand the phylogenetic and functional diversity of culturable cellulose degrading bacteria inhabiting the koala faeces using molecular and other biochemical methods.

## Methods

### Collection of sample

We didn’t use animals in this experiment. Koala faeces were obtained from Adelaide Zoo, South Australia with permission of Zoo authorities and no animal was harmed during the process. The faeces were collected by hand, using clean disposable gloves, and placed into a clean sterile plastic bag. The faecal pellets were lightly brushed to remove soil and the upper layer of these pellets was removed by scraping them with a sterile razor to detach any other foreign material.

### Isolation and enumeration of cellulose degrading bacteria

Bacteria were extracted from the faeces samples by blending 1 g wet wt of the sample with 9 ml of Tween buffer (1 % Tween 80 and 0.9 % NaCl) for 1 min before serial dilution. Bacterial strains were grown and isolated at 30 and 37 °C by plating on CMC-Congo red agar for enumeration of cellulose degraders by using the modified Hendricks method [13]. This medium contains 0.05 % K_2_HPO_4_, 0.025 % MgSO_4_, 0.188 % CMC sodium salt, 0.02 % Congo red, 0.02 % gelatine, 0.05 % yeast extract and 1.5 % agar. Morphologically different isolates with a halo zone around the colonies were selected and purified for genotypic and enzymatic studies.

### Qualitative screening of isolates for hydrolytic enzyme production and bioremediation potential

All isolates were screened for production of each enzyme on 1.8 % (w/v) agar plates containing one of the following substrates (supplied by Sigma-Aldrich, USA, unless otherwise stated): beech xylan (0.5 % w/v) for xylanases; CMC sodium salt (0.5 % w/v) for endoglucanases [14]; gelatin (1 % w/v) for proteases [15]; starch (1 % w/v) for amylases [16] and Rhodamine B lipase agar for lipases [17]. All plates contained minimal medium salts consisting of: KH_2_PO_4_ (1.5 % w/v), (NH_4_)_2_SO_4_ (0.5 % w/v), MgSO_4_.7H_2_O (0.06 % w/v) and CaCl_2_.2H_2_O (0. 06 % w/v). Tannase activity was detected employing the modified method devised by Osawa [5] in which nutrient agar was used in place of brain heart infusion agar. Hydrolysis capacity (HC) index for all ten isolates was calculated by dividing the diameter of the clear zone around the colony with bacterial colony diameter [18].

To evaluate the bioremediation potential, all cultures were grown in 50 ml mineral medium [19] supplemented with 100 ppm phenanthrene under shaking conditions (140 rpm) at 30 °C for 7 days. To raise inoculums, all the isolates were grown in Luria broth at 30 °C for 24 hrs, centrifuged (6000 rpm for 5 min) and pellet was washed three times with normal saline. Pellet was resuspended in normal saline and 2 ml inoculum (OD_600_ 0.8) was added to each flask. Any increase in OD_600_ was taken as an indication of bacterial growth and bioremediation potential [20].

### Genomic DNA extraction and 16S rRNA gene PCR amplification

Genomic DNA was extracted from all the isolates using the ultra clean microbial DNA isolation kit (Mo Bio, USA), following the manufacturer’s protocol. DNA preparations were visualized after electrophoresis in a 1.0 % agarose gel in 1x TBE buffer to assess their integrity and then stored at −80 °C prior to PCR amplification.

PCR amplification of 16S rRNA gene sequencing of isolated bacteria was done using universal primer pair consisting of 8 F (AGAGTTTGATCCTGGCTCAG) and 1541R (AAGGAGGTGGATCCANCCRCA) [21]. The final volume consisting of the reaction mixture of 50 μl contained 10 μl of Go Taq Buffer, 2.5 μl of MgCl_2_ (25 mM), 1 μl of dNTP, 1 μl of each primer, 2 μl (500 ng) template DNA and 0.25U of Taq polymerase. The amplification was done on Bio-Rad My cycler (Initial denaturation at 94 °C for 5 min followed by 32 cycles of 94 °C for 1 min, 56 °C for 1 min and extension at 72 °C and final extension at 72 for 10 min).

### DNA sequencing and phylogenetic analysis

DNA sequencing of the purified PCR product was carried out by SouthPath at Flinders Sequencing Facility, Flinders Medical Centre in Adelaide using Sanger’s di-deoxy nucleotide sequencing method. The sequences were compared with those in the GenBank database using the BLAST search program (http://www.ncbi.nlm.nih.gov/). Phylogenetic analysis was performed with the MEGA 5.0 program (Molecular Evolutionary Genetics Analysis, Version 5.0) [22]. The tree topologies were evaluated using bootstrap analyses based on 1000 replicates [23] and phylogenetic trees were inferred using the neighbour-joining method [24].

## Results

The population of cellulose degraders was 4.4 × 10^8^ cfu per gram of koala faeces. A total of 10 morphologically different colonies were selected from the highest dilution for the diversity studies.

### Qualitative screening of isolates for hydrolytic enzyme production and bioremediation potential

**Fig. 1:**
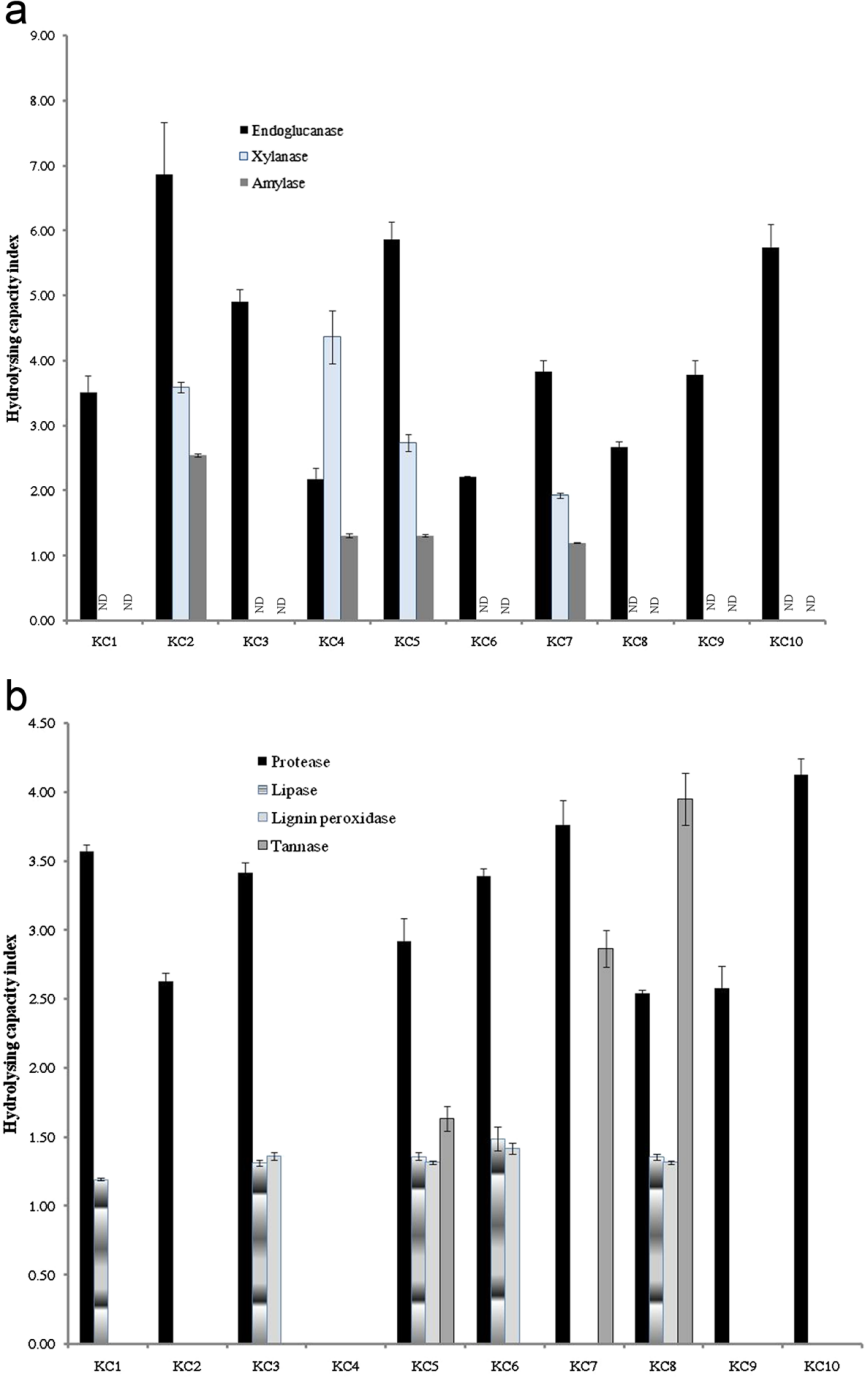
Hydrolyzing capacity index of the bacterial isolates on agar plates containing the substrate for the target enzyme. **a** endoglucanase, xylanase and amylase **b** Protease, Lipase, lignin peroxidise and tannase. All the assays were carried out at 25 °C and 48 h incubation except for lignin peroxidise and tannase (120 h). Error bars indicate standard deviation above and below the mean (*n* = 3)

Extracellular enzyme profiles of all the isolates revealed that besides endoglucanase, 40 % of isolates indicated xylanase, lignin peroxidise and amylase activities. The HCI for all the isolates were calculated and emerged that the isolate KC2 as well as KC4 and KC8 were good producers of endoglucanase (HCI 6.86), xylanase (HCI 4.37) and tannase (HCI 3.95), respectively (Fig. 1a). All the isolates except KC4 demonstrated proteolytic activities with a maximum HCI of 4.12 by KC10 isolate KC10. Besides protease, lipase, lignin peroxidise and tannase activities were also absent in isolate KC4 (Fig. 1b). Eight cultures were able to use phenanthrene as the sole carbon source and grew convincingly well after 7 days of incubation (Table 1).

**Table 1:** Utilization of phenanthrene (100 ppm) by different isolates

Isolate		Growth (OD 600 nm)
KC1	+	0.23
KC2	++	0.76
KC3	+	0.20
KC4	-	0.00
KC5	+	0.10
KC6	-	0.00
KC7	++	0.63
KC8	+	0.28
KC9	+	0.31
KC10	+	0.21

Growth was checked by measuring the increase of OD600 nm for 7 days

++: OD600 nm >0.5, +: OD600 nm >0.1, −: No growth

### Molecular characterization and phylogenetic analysis of isolates

**Fig. 2:**
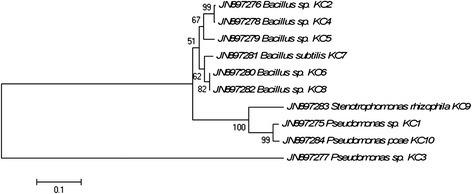
Phylogenetic tree based on the 16S rRNA gene sequences of different isolates and their closest phylogenetic relatives

Sequencing of the 16S rRNA gene was done using Sanger’s di-deoxy nucleotide sequencing method and the cultures were identified based on percentage similarity (>97 % compared with public database sequences, NCBI) *via* blastn search (http://www.ncbi.nlm.nih.gov) (Table 2). Out of total ten cultures, 6 belonged to the Bacillus genus while 4 were from phylum gamma proteobacteria and *Stenotrophomonas* genus. All sequences were submitted to NCBI GeneBank with the accession numbers ranging from JN897275 to JN897284. Neighbour-joining phylogenetic tree indicated that isolates formed coherent clusters with the closet relative available in Genbank public database that exhibited maximum similarity at 16S rRNA gene sequence (Fig. 2).

**Table 2:** Phylogenetic affiliations of culturable bacteria isolated from koala faeces

Isolate number	Accession number	Specifies Identified	Nearest neighbour	Similarity (%)
KC1	JN897275	*Pseudomonas sp.*	*Pseudomonas* sp. (HQ727967.1)	99
KC2	JN897276	*Bacillus sp.*	*Bacillus* sp. (JN208196.1)	100
KC3	JN897277	*Pseudomonas sp*	*Pseudomonas* sp. (HQ727967.1)	99
KC4	JN897278	*Bacillus sp.*	*Bacillus* sp. JG-TB2 (FR849914.1)	100
KC5	JN897279	*Bacillus sp*	*Bacillus* sp. DG7 (JN208198.1)	100
KC6	JN897280	*Bacillus sp.*	*Bacillus* sp. MX47 (JN578480.1)	100
KC7	JN897281	*Bacillus subtilis*	*Bacillus subtilis* (JN587510.1)	100
KC8	JN897282	*Bacillus sp.*	*Bacillus* sp. JN578480.1	100
KC9	JN897283	*Stenotrophomonas rhizophila*	*Stenotrophomonas rhizophila* strain PCA_13 (JF711015.1)	99
KC10	JN897284	*Pseudomonas poae*	*Pseudomonas poae* strain P 527/13 (NR_028986.1)	99

## Discussion

Over the last few decades, 2nd generation biofuels using lignocellulosic feed stocks have received considerable scientific attention due to rising crude oil prices and environmental concerns. Researchers are looking for new isolates with resilient cellulase enzyme system that can reduce the cost of saccharification involved in cheap biofuel production. Digestive tracts of herbivorous animals have always attracted researchers as potential sources of bacteria with unique properties [25–27] and many new promising microorganisms have been isolated from animal faeces [28, 29]. The koala of all herbivorous animals has peculiar dietary habits in that it feeds exclusively on eucalyptus leaves high in recalcitrant constituents like cellulose, lignin, essential oils and tannin. Cellulose degrading bacteria play an important role in the enzymatic degradation of cellulose present in eucalyptus leaves for releasing the nutrients for energy. While reports concerning the microflora of koala intestine specially tannin-protein degrading microflora are available, there is still a paucity of data on cellulose degrading bacteria from koala faeces.

To our knowledge, this is the first report of cellulose degrading bacteria from koala faeces or gastrointestinal tract. CMC hydrolysis is often used as a test for endoglucanase and β-glucosidase activity [30]. CMC assay for cellulose degradation is well established and has been used by different workers with or without modifications as an indicator for cellulose degradation [14, 31, 32]. However, all these methods are time-consuming and use either precipitating agents like hexadecyltrimethyl ammonium bromide (HAB) or congo red for flooding the plates to detect the zone of hydrolysis, which makes isolation of the effective isolates very difficult from the same plates. In the present study, we used modified medium in which CMC and congo red was incorporated in the enumeration medium and the cellulose degrading colonies can be clearly distinguished by the presence of zones of clearing from those that did not use CMC.

In the present study, a considerable population of the cellulose degraders was isolated from the koala faeces and interestingly all the cellulose degraders were either from phylum firmicutes (*Bacillus*) or gamma proteobacteria based on the blast analysis. All the isolates were assigned to respective species by comparing the closet relative from NCBI database. Gamma proteobacteria and firmicutes are among the most commonly isolated bacterium from faecal and intestinal samples of various animals [33–36]. *Bacillus* and related spp. are also routinely isolated from animal faeces and intestine samples of many vertebrates [33–40]. Similarly, gamma proteobacteria constitute the major population in the gastrointestinal tract and faeces of animals [41–44]. In this analysis all the isolates emanated from these classes with the majority being the *Bacillus* spp. Interestingly, *Pseudomonas poae*, a fluorescent bacterium reported earlier from the phyllosphere of grasses [45] was also present in koala faeces. It may have been ingested by the koala as part of their diet and the bacterium adapted to the gastrointestinal environment forming a symbiotic association with koala.

In addition to cellulase activities, protease, amylase, lipase and tannase activities were also tested for all the isolates. Hydrolysing capacity index used in the study provided a semi-quantitative measure of these bacteria’s enzyme production. Most isolates showed more than one extracellular enzyme activity which might be acting synergistically to hydrolyse the toxic eucalyptus leaves in the koala intestine. These enzymes produced by the isolates have various industrial applications. Xylanases are used in paper and pulp production to reduce the use of toxic chlorine compounds that increase pulp brightness [46]. Similarly, amylases are employed in many starch conversion, food and textile industries [47] while tannases have potential to be used in the tea and wine industries [48]. Proteases and lipases have been employed in the textiles, dairy and leather industries in recent decades [49, 50].

Many isolates were able to grow in phenanthrene (100 ppm) which is considered to be toxic and carcinogenic compounds that are ubiquitous pollutants in the environment [51]. The capacity to degrade a ring compound like phenanthrene in some cultures might be due to the presence of lignin peroxidase enzyme system which can breakdown a number of pollutants like PAH due to their non-specific action [52]. In other cultures some other pathway of degradation may be involved [53]. These isolates can be exploited further for the bioremediation of other related PAH compounds.

## Conclusion

To conclude, the cellulase degraders isolated from koala faeces have revealed many hydrolytic enzymes that have industrial potential besides cellulase. Furthermore the isolates also possess the ability to grow in the presence of pollutants like phenanthrene, indicating their possible application for bioremediation. Potential isolates will be used in further studies to characterise the enzymes and genes for industrial and bioremediation applications.
